# Chromosomal Mapping of Tandem Repeats Revealed Massive Chromosomal Rearrangements and Insights Into *Senna tora* Dysploidy

**DOI:** 10.3389/fpls.2021.629898

**Published:** 2021-02-10

**Authors:** Nomar Espinosa Waminal, Remnyl Joyce Pellerin, Sang-Ho Kang, Hyun Hee Kim

**Affiliations:** ^1^Department of Chemistry and Life Science, BioScience Institute, Sahmyook University, Seoul, South Korea; ^2^Genomics Division, National Institute of Agricultural Sciences, Rural Development Administration, Jeonju, South Korea

**Keywords:** satellite DNA, *Senna tora*, tandem repeats, dysploidy, fluorescence *in situ* hybridization, whole genome duplication, karyotype

## Abstract

Tandem repeats can occupy a large portion of plant genomes and can either cause or result from chromosomal rearrangements, which are important drivers of dysploidy-mediated karyotype evolution and speciation. To understand the contribution of tandem repeats in shaping the extant *Senna tora* dysploid karyotype, we analyzed the composition and abundance of tandem repeats in the *S. tora* genome and compared the chromosomal distribution of these repeats between *S. tora* and a closely related euploid, *Senna occidentalis*. Using a read clustering algorithm, we identified the major *S. tora* tandem repeats and visualized their chromosomal distribution by fluorescence *in situ* hybridization. We identified eight independent repeats covering ~85 Mb or ~12% of the *S. tora* genome. The unit lengths and copy numbers had ranges of 7–5,833 bp and 325–2.89 × 10^6^, respectively. Three short duplicated sequences were found in the 45S rDNA intergenic spacer, one of which was also detected at an extra-NOR locus. The canonical plant telomeric repeat (TTTAGGG)_n_ was also detected as very intense signals in numerous pericentromeric and interstitial loci. StoTR05_180, which showed subtelomeric distribution in *Senna occidentalis*, was predominantly pericentromeric in *S. tora*. The unusual chromosomal distribution of tandem repeats in *S. tora* not only enabled easy identification of individual chromosomes but also revealed the massive chromosomal rearrangements that have likely played important roles in shaping its dysploid karyotype.

## Introduction

Numerous genome studies have shown the ubiquity and abundance of repetitive elements in plant genomes and have validated the crucial role of repeats in genome structure, function, and evolution (Wicker et al., 2007; Shatskikh et al., 2020). Once notoriously labeled as “junk DNA,” repetitive elements are now known as important players in gene regulation, stress response, and genome stability (Fedoroff, 2012). However, the same repeats are also substrates for, and byproducts of, chromosomal rearrangements, thereby driving variations in chromosome structure between closely related lineages (Schubert and Lysak, 2011; Murat et al., 2017). Tracing the dynamics of different repeat families among closely related taxa could therefore provide insights into a genome's evolutionary past (Long et al., 2013; Waminal et al., 2018b).

Repetitive elements are classified into two major types: dispersed repeats and tandem repeats. Dispersed repeats, such as transposable elements, are scattered either sparsely or extensively in one or more genomic loci without following a head-to-tail organization (Frello and Heslop-Harrison, 2000). In contrast, tandem repeats follow a head-to-tail organization in one or more genomic loci. They include microsatellites (2–5 bp of usually 10–100 units), minisatellites (6–100 bp of 0.5–30 kb arrays), and longer satellites with varied unit lengths (usually 150–400 bp) and array size of up to several megabase pairs (Mehrotra and Goyal, 2014; Garrido-Ramos, 2017).

Tandem repeats are abundant in heterochromatic regions of chromosomes, such as the (peri)centromere and (sub)telomere. Although a single satellite family often dominates either of these chromosomal regions (Garrido-Ramos, 2017), many other repeats occupy these sites at low copies forming a library of repeats that could amplify at any favorable time, such as in response to genomic shock (Fedoroff and Bennetzen, 2013; Ruiz-Ruano et al., 2016). Moreover, ectopic (non-allelic) (micro)homologies between the (peri)centromere and (sub)telomere make these regions hotspots for chromosomal rearrangements (Salser et al., 1976; Kubis et al., 1998; Schubert and Lysak, 2011; Pellestor and Gatinois, 2018; Rosato et al., 2018; Hartley and O'Neill, 2019).

Chromosomal rearrangements may occur either intrachromosomally via segmental deletion, addition, or inversion, or interchromosomally via arm translocation, telomere-telomere “fusions” (also known as end-to-end translocation [EET]), and nested chromosome insertion (NCI) (Mandáková et al., 2019). Interchromosomal rearrangements can generate dysploid karyotypes, that is, altered chromosome numbers (Murat et al., 2010; Schubert and Lysak, 2011; Mandáková and Lysak, 2018). When chromosomes are fragmented and the fragments maintain or develop centromere function, species with increased chromosome numbers (ascending dysploid) are produced (Rousselet et al., 2000; Chung et al., 2011; Mandáková and Lysak, 2018; Schubert et al., 2020). Conversely, EET and NCI could generate species with fewer chromosomes (descending dysploid) (Rousselet et al., 2000; Mandáková and Lysak, 2018). For example, EETs have been implicated in the karyotype evolution of the ant *Myrmecia pilosula* (Imai and Taylor, 1989), human (Ijdo et al., 1991), and many crucifers (Mandáková et al., 2017), whereas NCI is more prevalent in grasses (Luo et al., 2009; Murat et al., 2010). Moreover, dysploidy, particularly descending dysploidy, is more prevalent in angiosperms than originally thought (Sousa and Renner, 2015; Levin, 2020).

Although tandem repeats have the potential to generate chromosome rearrangements, they could also be a product of such rearrangements, that is, tandem repeats and chromosomal rearrangements are closely associated (Sousa and Renner, 2015; Louzada et al., 2020). Tandem repeats can also undergo massive amplification through concerted evolutionary processes, including unequal crossing over, replication slippage, gene conversion, or rolling circle replication of circular DNA (Charlesworth et al., 1994; Cohen and Segal, 2009; Rosato et al., 2018). Sometimes, novel repeat families arise after re-establishing genome stability from stressful events, such as a whole-genome duplication (WGD) (Fedoroff, 2012), which sometimes generates new taxonomic lineages (Murat et al., 2010; Waminal et al., 2016). Because the fixation of tandem repeats in terms of abundance and chromosome location may vary across different taxa in a clade (Perumal et al., 2017), quantifying and chromosomal mapping of tandem repeats can provide useful data to understand genome history and species relationships.

The genus *Senna* (Family Leguminosae, Subfamily Caesalpiniaceae) comprises many anthraquinone-producing medicinal plants (Jang et al., 2007; Puri, 2018; Kang et al., 2020b). Specifically, *Senna tora* L. (Roxb) (syn. *Cassia tora* L.) has been used traditionally as a dying agent, tea, or herbal medicine in several Asian countries, such as India, China, and Korea, and is now gaining global attention, prompting its genome sequencing initiative (Puri, 2018; Kang et al., 2020a).

The predominant diploid chromosome number in *Senna* is 2*n* = 28. However, species with descending dysploid karyotypes of 2*n* = 22–26 have also been reported, including *S. tora* with 2*n* = 26 chromosomes (Pellerin et al., 2019). The taxonomic classification of *S. tora* and its related species is still ambiguous (Marazzi et al., 2006), and although the genome of *S. tora* has been released recently, no comprehensive comparative genomics with related *Senna* species has been performed to understand its genome evolution (Kang et al., 2020a). Nevertheless, analyzing the abundance of repeats using short next-generation sequencing reads and their chromosomal distribution through fluorescence *in situ* hybridization (FISH) could provide relevant insights into the karyotype evolution and genome history of *S. tora* (Ruiz-Ruano et al., 2016; Novák et al., 2017).

Here, we performed *in silico* mining for high-abundance tandem repeats in the *S. tora* genome using 0.1× whole-genome short reads. We then visualized the chromosomal distribution through FISH using repeat-specific pre-labeled oligo probes (PLOP) to understand the composition and role of tandem repeats in *S. tora* karyotype evolution. Unconventional chromosomal distribution of *S. tora* tandem repeats, especially when compared with its closely related species, *Senna occidentalis*, provided cytogenetic evidence of past extensive chromosomal rearrangements that may have shaped the extant *S. tora* genome.

## Materials and Methods

### Plant Samples

*S. occidentalis* and *S. tora* seeds were kindly provided by the Department of Herbal Crop Research, NIHHS, RDA, Eumseong 369–873, South Korea. Seeds were germinated in potting soil and incubated in the greenhouse at 24–26°C. Root tips were treated with 2 mM 8-hydroxyquinoline for 4 h, fixed with aceto-ethanol (1:3 v/v), and stored in 70% ethanol at 4°C until use.

### Chromosome Spread Preparation

Sporophytic metaphase chromosome slide preparations were performed according to the technique of Waminal et al. (2012) with some modifications. Briefly, meristematic tips (~2 mm) were digested in a 50 μl pectolytic enzyme solution (2% Cellulase RS [Duchefa, Haarlem, The Netherlands, C8003.001] and 1% Pectolyase Y-23 [Duchefa, P8004.0001] in 100 mM citrate buffer) for 2 h at 37°C, and washed with distilled water. Roots were then transferred into a microtube containing chilled Carnoy's solution and vortexed for 30 s at room temperature. After disposing of the supernatant, the pellet was resuspended in (9:1 v/v) aceto-ethanol. The cellular suspension was then pipetted onto clean glass slides prewarmed in a humid chamber. After air-drying, slides were fixed for 5 min in 2% formaldehyde (Vrana et al., 2012), quickly dipped into distilled water, and dehydrated using ascending concentrations of ethanol (70, 90, and 100%).

### Repeat Mining and Quantification

Paired-end reads (100 bp), about 2.9× of the 686 Mb *S. tora* genome (Ohri et al., 1986), were obtained from the International Cooperation Team, International Technology Cooperation Center, RDA, Jeonju 54875, South Korea. Read quality trimming, read sampling, and repeat clustering were carried out using TAREAN (Novák et al., 2017). All consensus sequences were generated by TAREAN except for the Sto_5S rDNA, StoIGS_463, StoIGS_293, and StoIGS_188. The 5S rDNA contig was identified from dnaLCW analysis (Kim et al., 2015) using CLC Genomics Workbench (CLC Inc., Aarhus, Denmark) and annotated by BLAST against the 5S rDNA database (Szymanski et al., 2016). StoIGS_463, StoIGS_293, StoIGS_188, and other repeats in the IGS of 45S rDNA were identified using Tandem Repeats Finder (Benson, 1999) and BLAST (Altschul et al., 1990). Tandem repeats identified with TAREAN were named following a similar nomenclature as Ruiz-Ruano et al. (2016). The four 45S rDNA contigs generated by TAREAN were assembled manually using the CLC Genomics Workbench. Sequences were submitted to Genbank and accession numbers are shown in corresponding tables.

We quantified the copy number and genome proportion of each repeat by mapping 2.7× trimmed short reads to the consensus sequences of the tandem repeats using CLC Genomics Workbench (CLC Inc.) and normalized the values to the 686-Mb 1C genome size of *S. tora*. For the 45S rDNA, we used only the 5,833 bp coding region without the IGS for quantification because of the different repeats in the IGS that could distort quantification. Short repeats were concatenated to form longer arrays for efficient read mapping.

### Probe Design

To visualize the chromosomal distribution of the repeats, we designed pre-labeled oligonucleotide probes (PLOPs). PLOPs for StoTR01_86, StoTR03_178, StoTR04_55, StoTR05_180, StoTR06_159, and StoIGS_463 were designed using the CLC Main Workbench and were synthesized by Bioneer (Daejeon, South Korea). PLOPs previously developed for the coding regions of the 5S rDNA and 45S rDNA, and the *Arabidopsis*-type telomeric repeat, which has the same sequence as StoTR02_7_tel, were used to localize these repeats (Waminal et al., 2018a). Details of the designed PLOPs are summarized in Supplementary Table 1.

### FISH and Karyotyping

A rapid FISH method was performed for PLOP-labeled probes (Waminal et al., 2018a). For PCR amplicon probes labeled via nick-translation, FISH was performed according to a modified procedure of Waminal et al. (2020). Homologous chromosomes were identified based on their FISH signals, morphological characteristics, and lengths considering previous karyotype data for *S. tora* (Pellerin et al., 2019). Karyograms were created using Adobe Photoshop CS6, whereas the idiogram and other diagrams were generated using Adobe Illustrator CS6.

## Results

### Tandem Repeats With Various Lengths Comprise >12% of the *S. tora* Genome

**Figure 1:**
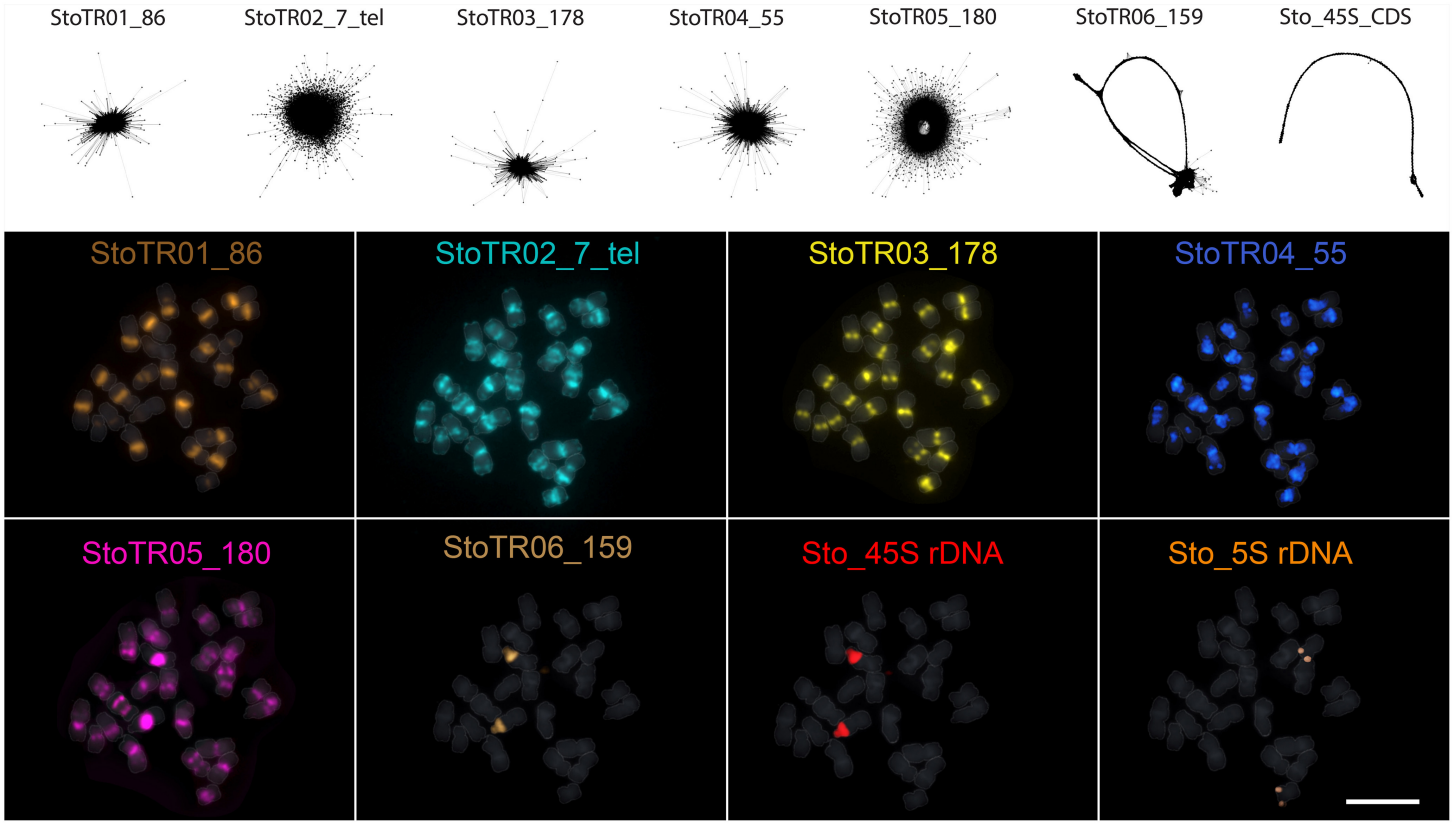
Independent repeats identified through read clustering of <1 × *S. tora* genome. The upper panel shows the read cluster graphs of the seven tandem repeats identified through TAREAN. No cluster data were generated for the 5S rDNA, which was identified using dnaLCW. The lower panel shows the chromosomal distribution of the eight repeats from the upper panel and the 5S rDNA. Bar = 10 μm.

A total of eight tandem repeats were identified using both TAREAN and dnaLCW (Table 1). Short read clustering of approximately 0.1× of the 686 Mb *S. tora* genome (Ohri et al., 1986), using TAREAN, generated seven satellite consensus sequences (Figure 1, Table 1). Of these, three were minisatellites: the canonical (*Arabidopsis*-type) plant telomeric minisatellite (TTTAGGG)_n_, which we named StoTR02_7_tel, StoTR01_86, and StoTR04_55. Three were satellites (StoTR03_178, StoTR05_180, and StoTR06_159), and one is the 45S rDNA coding sequence (Sto_45S_CDS).

**Table 1:** Independent tandem repeats identified in the *S. tora* genome.

**No**	**Name**	**Analysis tool**	**Length (bp)**	**AT (%)**	**Copy no**.	**Length (kb)^a^**	**GP (%)^b^**	**Accession no**.
1	StoTR01_86	TAREAN	86	65	313,414	26,953.6	3.93	MW143025
2	StoTR02_7_tel	TAREAN	7	57	2,888,813	20,221.7	2.95	MW143031
3	StoTR03_178	TAREAN	178	49	85,581	15,233.4	2.22	MW143027
4	StoTR04_55	TAREAN	55	71	194,692	10,708.1	1.56	MW143024
5	StoTR05_180	TAREAN	180	58	13,772	2,478.9	0.36	MW143028
6	StoTR06_159	TAREAN	159	47	14,432	2,294.7	0.33	MW143026
7	Sto_45S_CDS	TAREAN	5,833	45	1,225	7,148.2	1.04	MW143033
8	Sto_5S	dnaLCW	181	48	325	87.4	0.01	MW143032

a *Length (kb) = copy number × repeat length/1,000*.

b *Genome Proportion = length/S. tora genome size (686 Mb) × 100*.

**Figure 2:**
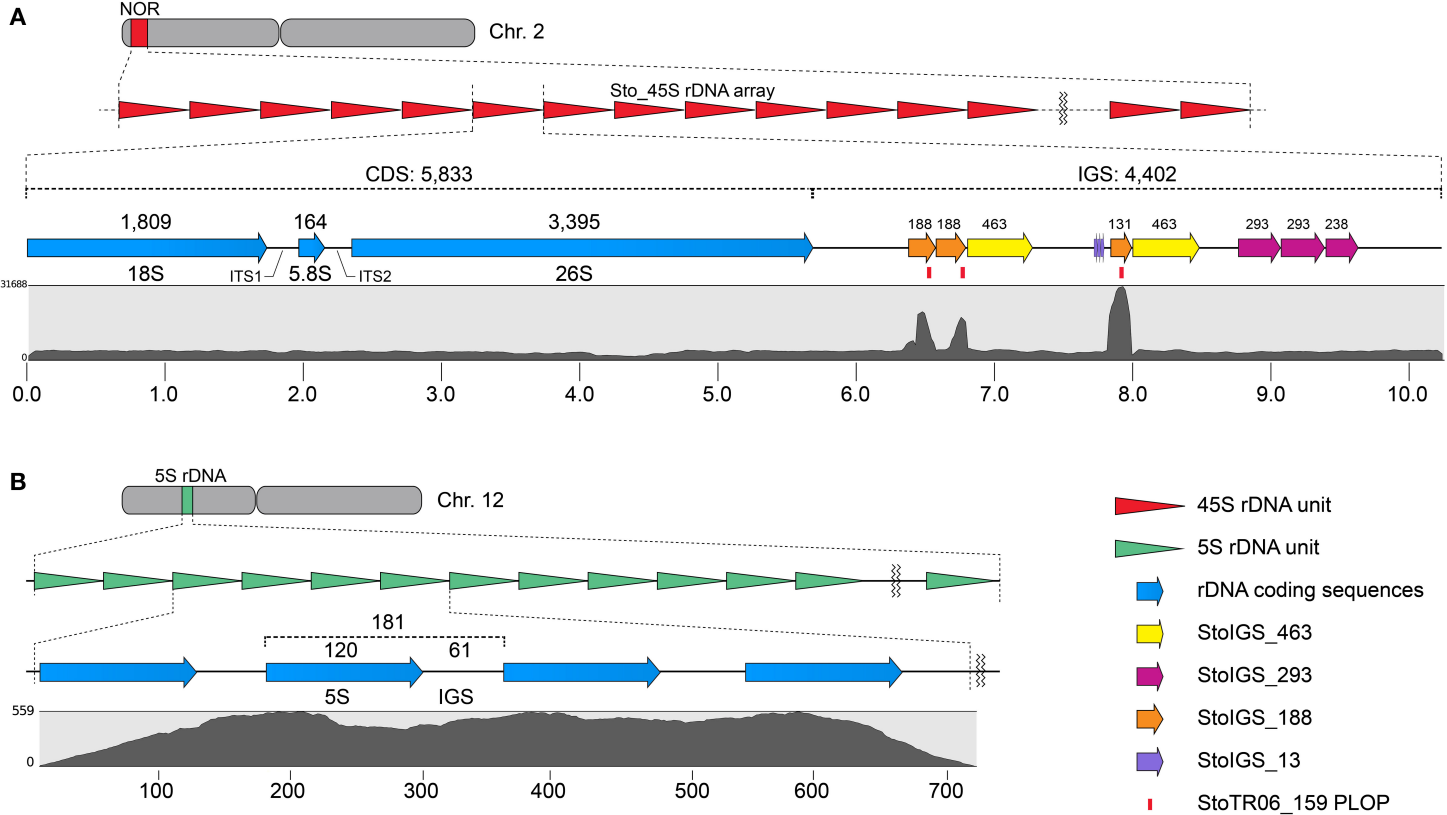
Sequence organization of the 45S **(A)** and 5S **(B)** rDNA sequences in *S. tora*. **(A)** 10,235 bp *S. tora* 45S rDNA consensus sequence in chromosome 2. The StoIGS_463 (yellow arrows) was localized immediately downstream of the StoIGS_188 repeat arrays. Mapping of 2.7× short reads (gray graph) revealed an extreme mapping abundance of the StoIGS_188 coincidentally matching with the StoTR06_159 PLOPs (red bars). **(B)** 181 bp 5S rDNA in chromosome 12. The numbers on top indicate the bp lengths of corresponding fragments. Scales in **(A)** and **(B)** are in kb and bp, respectively.

Three other clusters from the TAREAN output were grouped with the Sto_45S_CDS into a single 45S rDNA supercluster. Assembling these clusters generated a 10,235 bp 45S rDNA consensus with a 5,833 bp coding sequence (CDS) and a 4,402 bp intergenic spacer (IGS, Figure 2A, Table 1). The TAREAN pipeline was not able to generate any cluster associated with the 5S rDNA, indicating a low copy number in the *S. tora* genome. Therefore, to assemble the 5S rDNA sequence, we performed a *de novo* assembly of 2.7× short reads using the dnaLCW method (Kim et al., 2015). This approach enabled the identification of a 181 bp 5S rDNA contig, Sto_5S (Figure 2B, Table 1).

All eight tandem repeats represented 0.01~ 3.93%, totaling 12.4%, of the *S. tora* genome (Table 1). In terms of total physical length, five repeats covered >7 Mb or >1% genome proportion (GP) (Table 1). StoTR01_86 covered ~27 Mb or 3.9% GP followed by StoTR02_7_tel (~20 Mb, 2.7%), StoTR03_178 (~15.2 Mb, 2.2%), StoTR04_55 (~10.7 Mb, 1.6%), and Sto_45S_CDS (~7.1 Mb, 1.0%). Because of the various repeats in the 45S rDNA IGS which could potentially distort quantification (see below), we only used the CDS for 45S rDNA length and GP estimation. The actual values are expected to be higher. The other three repeats each had <1% GP, of which Sto_5S had the lowest values for length and GP at 87.4 kb and 0.01%, respectively.

Shorter REs tended to have higher copy number. The shortest repeat, StoTR02_7_tel, showed the highest copies at 2.89 × 10^6^ followed by StoTR01_86 (3.13 × 10^5^), StoTR04_55 (1.92 × 10^5^), StoTR03_178 (8.56 × 10^4^), StoTR06_159 (1.44 × 10^4^), and StoTR05_180 (1.38 × 10^4^). However, the short 5S rDNA which was only 181 bp was much fewer than the 5,833 bp 45S rDNA (325 vs. 1,225 copies, respectively).

### Short Duplicated Sequences Were Present in the Intergenic Spacer of *S. tora* 45S rDNA

We analyzed for the presence of duplicated sequences in the 4,402-bp 45S rDNA IGS region, considering that this region often carries different repeats and is known to be involved in genome reorganization in some species (Elliott et al., 2013; Havlová et al., 2016). We identified three >100 bp short duplicated sequences (Figure 2A, Table 2). The longest was 463 bp long (StoIGS_463) and was present in two copies but not tandemly arranged. The second was 293 bp long (StoIGS_293) and was present in tandemly arranged 2.8 copies toward the 3′ end of the IGS. The third was 188 bp long (StoIGS_188) and was also tandemly arranged and distributed into two disjunct sites, each immediately upstream of the StoIGS_463. The upstream and downstream sites each had 2.0 and 0.6 copies of the StoIGS_188. A short array of a 13 bp tandem repeat, StoIGS_13, was also localized upstream of the 0.6-copy StoIGS_188 locus.

**Table 2:** Short duplicated sequences identified in the 45S rDNA intergenic spacer using Tandem Repeats Finder.

**No**	**Name**	**Type**	**Length (bp)**	**AT (%)**	**Copy no**.	**Length (kb)^a^**	**GP (%)^b^**	**Accession no**.
1	StoIGS_463	IGS-related	463	65	2,612	1,209.2	0.18	MW143034
2	StoIGS_293	IGS-related	293	45	3,292	964.7	0.14	MW143035
3	StoIGS_188	IGS-related	188	48	3,856	724.9	0.11	MW143036

a *Length (kb) = copy number × repeat length/1,000*.

b *Genome Proportion = length/S. tora genome size (686 Mb) × 100*.

Mapping of short reads to the 45S rDNA consensus sequence revealed a high coverage depth bias in the StoIGS_188 regions (Figure 2A), which is indicative of either a collapsed IGS region in the consensus sequence or an extra-IGS homologous locus.

### StoTR06_159 Is a Deletion Variant of StoIGS_188

**Figure 3:**
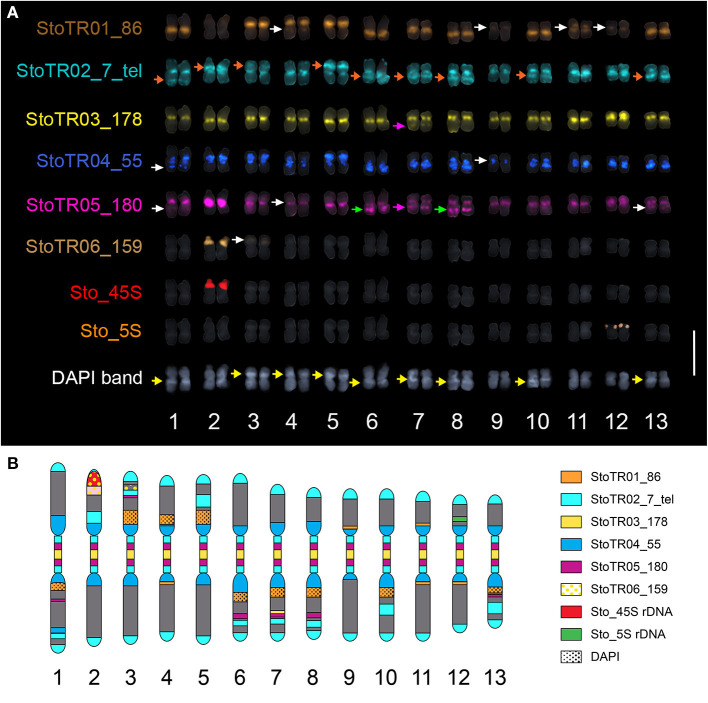
Mitotic metaphase FISH karyogram of *S. tora***. (A)** Chromosomal distribution of the eight independent tandem repeats showing mostly centromeric and pericentromeric localization. StoTR01_86 localized at paracentric regions of either short or long arms, except for chromosomes 4 and 11 which showed pericentric localization. StoTR02_7_tel showed extra-telomeric signals at the centromere and interstitial regions of either, but not both, chromosome arms (orange arrows). StoTR03_178 localized at centromeres of all chromosomes and at a weak locus in 7L which colocalized with StoTR05_180 (pink arrows). StoTR04_55 localized at pericentromeric regions in all chromosomes and a weak locus in 1L. StoTR05_180 was intense in the centromere of chromosome 2. Aside from centromeric regions, it was also observed at equilocal sites in the interstitial regions of some chromosomes (green, white, and pink arrows). StoTR06_159 showed an intense signal at the NOR site in chromosome 2S and a weak signal in chromosome 3S. One locus each was observed for the 45S and 5S rDNA families. Some chromosomes have inherent DAPI bands (yellow arrows). Note the colocalized StoTR06_159 and the NOR site in chromosome 2S. White arrows indicate weak signals. Bar = 10 μm. **(B)** Karyotype idiogram of *S. tora* with stretched (peri)centromeric region. Chromosomal niches of different repeats are indicated by different colors and patterns. The dark gray background indicates the DAPI counterstain.

Our FISH results for StoTR06_159 showed unexpected colocalization with the 45S rDNA locus (Figures 1, 3). Therefore, we mapped the StoTR06_159 PLOPs to the Sto_45S consensus sequence to identify regions of homology between StoTR06_159 and Sto_45S. Interestingly, the PLOPs mapped to the deep-coverage StoIGS_188 regions indicating sequence homology between StoIGS_188 and StoTR06_159 (Figure 2A). A sequence comparison between these two TRs revealed up to ~76% identity mostly caused by deletions in StoTR06_159 (Supplementary Figure 1). Three deletions were identified totaling 29 bp (9, 10, and 10 bp each) at 4–12, 41–50, and 82–91 nt. Outside these deletions, both TRs showed >87% identity.

### FISH Revealed Predominant (peri)centromeric Distribution of *S. tora* Tandem Repeats

Five of the eight tandem repeats (StoTR01_86, StoTR02_7_tel, StoTR03_178, StoTR04_55, StoTR05_180), which accounted for >11% GP, showed predominantly (peri)centromeric distribution (Figure 3A, Table 3). StoTR01_86, which had the highest genome proportions among all repeats, showed nine major and three minor loci. All major loci were exclusively paracentromeric at either the short (S) or long (L) arms of these nine chromosomes. The minor signal on Chr. 11 was pericentromeric, whereas those on chromosomes 4, 9, and 12 were paracentromeric (Table 3).

**Table 3:** Chromosomal distribution of the DNA repeats identified in the *S. tora* genome.

**No**	**Name**	**Loci^a^**	**Chromosomal distribution**
1	StoTR01_86	9/4	Major: paracentromeric on 9 chromosomes, 3 on short (S), and 6 on long (L) arms: (1L, 3S, 4S, 5S, 6L, 7L, 8L, 10L, and 13L). Minor: pericentromeric on chr. 11 and paracentromeric on 4L, 9S, and 12L
2	StoTR02_7_tel	13	Chromosome termini, pericentromeric in all chromosomes, interstitial on 1L, 2S, 3S, 5S, 6L, 7L, 8L, 10L, and 13L
3	StoTR03_178	13/1	Major: centromeric in all chromosomes, Chr. 12 more intense Minor: interstitial on 7L
4	StoTR04_55	12/2	Major: pericentromeric in all chromosomes except Chr. 9 Minor: pericentromeric in Chr. 9, interstitial on 1L
5	StoTR05_180	12/3	Major: centromere of Chr. 2 most intense, all centromeres except for chromosome 4, interstitial regions of 6L, 7L, and 8L Minor: centromere of chromosome 4, an interstitial region of 1L and 13L
6	StoTR06_159	1/1	Major: NOR site at 2S Minor: interstitial region of 3S
7	Sto_45S_CDS	1	2S
8	Sto_5S	1	12S
9	StoIGS_463	1	Colocalized at NOR site at 2S

a *Number of chromosomes bearing major/minor FISH signals*.

StoTR02_7_tel, an *Arabidopsis*-type telomeric repeat, was unusually abundant in the *S. tora* genome, in both repeat clustering quantification and FISH (Figure 3, Table 1). In addition to the canonical distribution at all chromosome termini, the StoTR02_7_tel FISH signals were intense in the pericentromeric region of all chromosomes. Although distinguishing the active centromere from the pericentromeric region was not straightforward in highly condensed metaphase chromosomes, we considered the StoTR02_7_tel loci as pericentromeric because active centromeres are unlikely to carry telomeric repeats, but instead have homogenous tandem repeats like those of StoTR03_178 (Hartley and O'Neill, 2019). Moreover, upon closer examination, StoTR02_7_tel signals do not perfectly coincide with those of StoTR_178 (Figure 3A). In addition to these pericentromeric loci, nine chromosomes carry roughly equilocal StoTR02_7_tel loci at either, but not both, short or long arms (2S, 3S, 5S, 1L, 6L, 7L, 8L, 10L, and 13L) (Table 3). Equilocality of repeats at interstitial regions in a chromosome are often relics of repeat displacement from the subtelomere (Garrido-Ramos, 2015).

StoTR03_178 showed two distinct FISH signals at all metaphase chromosome centromeres, one per sister chromatid (Figure 3). The signal in chromosome 12 was noticeably more intense than those in other centromeres, and a weak signal was observed in the interstitial region of chromosome 7L. Although we did not perform immunostaining for CENH3, the hallmark of an active centromere (Kursel and Malik, 2016), for lack of *S. tora*-specific CENH3 antibody, it is likely that StoTR03_178 could be involved in active centromere function because of its abundant copies and distinct FISH signals characterizing functional regional centromeres (Schubert et al., 2020). StoTR05_180 was detected in all chromosome pericentromeres and some interstitial regions. The centromeric signal in chromosome 2 was more intense than in other chromosomes. In addition to the centromeric loci, five equilocal interstitial loci were observed in five chromosomes: 1L, 6L, 7L, 8L, and 13L. Signals from 1L to 13L were weak and that of 7L was colocalized with StoTR03_178 (Figure 3).

StoTR04_55 localized to the pericentromeric region in all chromosomes. Except for chromosome 9, all chromosomes had intense signals. In addition, a minor signal was observed in the interstitial region of chromosome 1L. The dispersed distribution of StoTR04_55 is characteristic of pericentromeric retrotransposons found in other plants (Lim et al., 2007). StoTR06_159 was not localized at the centromere but revealed a colocalized signal at the NOR site of chromosome 2S and a weak signal at the interstitial region of chromosome 3S.

Both Sto_45S and Sto_5S rDNA had only one locus each at chromosomes 2S and 12S, respectively, as previously reported (Pellerin et al., 2019).

### FISH Revealed Specific Localization of StoIGS_463 at the 45S rDNA Locus

Because some repeats that have been identified in the 45S rDNA IGS region in *S. tora* and other plants have also been detected in other chromosomal loci via FISH [see StoTR06_159 and Lim et al. (2004)], we checked whether this is the same case for StoIGS_463, although we observed no evidence of biased abundance from read mapping (Figure 2A). Unlike StoIGS_188/StoTR06_159, which showed overabundance in read mapping and a weak FISH signal at chromosome 3S, StoIGS_463 did not show any extra-NOR signal, suggesting its specific localization in the 45S rDNA IGS (Supplementary Figure 2). Moreover, although we did not perform FISH with StoIGS_293, it is likely that it is also specific to the IGS based on the absence of depth variation in the read mapping similar to that of StoIGS_463 (Figure 2A).

### StoTR03_178 Is a More Abundant Centromeric Sequence Variant of StoTR05_180

Centromeric repeats are often categorized into families, which share >80% identity between members, and superfamilies with <80% identity with other families (Lim et al., 2007; Ruiz-Ruano et al., 2016; Hartley and O'Neill, 2019). We compared the consensus sequences of StoTR03_178 and StoTR05_180 to see whether they are evolutionarily related. StoTR03_178 and StoTR05_180 shared ~71% sequence identity, suggesting that these are two separate families in the same *S. tora* centromeric superfamily (Supplementary Figure 3). Moreover, sequences homologous to CENP-B box (CENP-B box-like), which play important roles in centromere function (Okada et al., 2007), were detected in both StoTR03_178 and StoTR05_180 (Supplementary Figure 3). However, only StoTR03_178 carries a 7 bp palindromic dyad symmetry region, which could indicate active centromeric DNA (Kasinathan and Henikoff, 2018).

Moreover, StoTR03_178 was ~6-fold more abundant than StoTR05_180 in *S. tora* (Table 1). Although both showed centromeric distribution, StoTR03_178 was more exclusively localized in the centromeres, whereas StoTR05_180 was abundant in pericentromeric and interstitial regions (Figure 3, Table 3). In addition, while the overall identity between the two families was <80%, one StoTR05_180 FISH probe, StTR180_OP2, was ~82% identical with StoTR03_178 (Supplementary Figure 3). This probe could not distinguish between StoTR03_178 and StoTR05_180 targets, which could explain the seemingly abundant StoTR05_180 FISH loci compared with the *in-silico* quantification data (Table 1). Nevertheless, non-centromeric signals from StoTR05_180, particularly at the interstitial regions of 1L, 6L, 7L, 8L, and 13L, along with the intense centromeric signal on chromosome 2, indicate the presence of cytologically exclusive niches occupied by the StoTR05_180 family.

### Comparative FISH With *S. occidentalis* Revealed Extensive Chromosomal Rearrangements in *S. tora*

**Figure 4:**
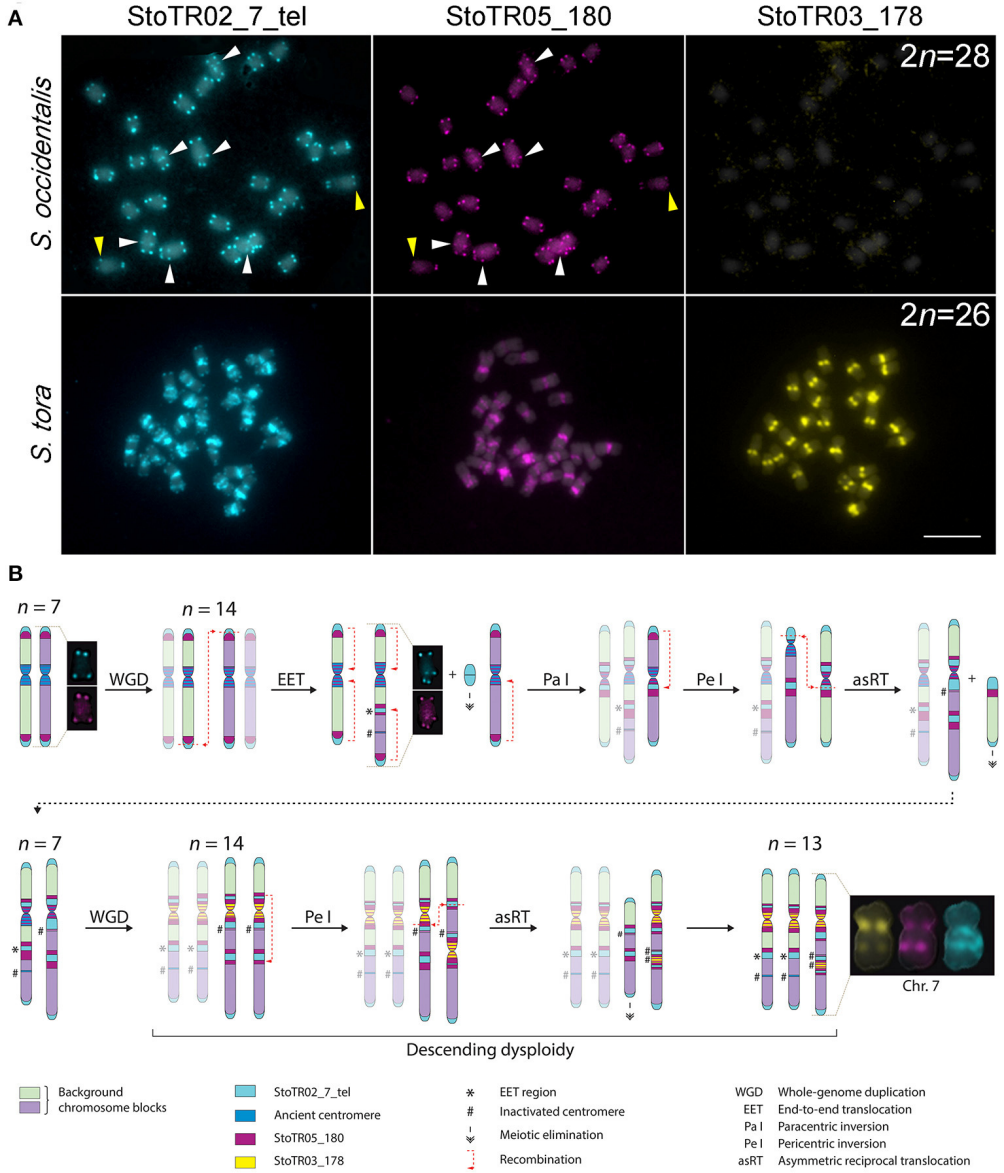
Hypothetical pathway for chromosome dysploidy in *S. tora***. (A)** Comparative FISH of StoTR02_7_tel, StoTR05_180, and StoTR03_178 between *S. occidentalis* and *S. tora*. StoTR02_7_tel and StoTR05_180 colocalized at chromosome termini and interstitial regions (white arrowheads) in *S. occidentalis* with some chromosomes showing differential abundance (yellow arrowheads). Both repeats were localized at pericentric and interstitial regions in *S. tora*. StoTR03_178 was observed only in *S. tora*. Bar = 10 μm. **(B)** Hypothetical chromosome evolution in *S. tora* attempts to explain the amplification of and StoTR02_7_tel and StoTR05_180 at pericentromeric and interstitial sites and the emergence of a novel StoTR03_178 centromeric repeat. Dysploidy and non-dysploidy mechanisms revert the chromosome number to *n* = 7 after a WGD. A second, more recent WGD triggers concerted evolution of StoTR03_178, displacing StoTR05_180 distally from the centromere. Two chromosomes merged forming a derivative chromosome (Chr. 7) causing the extant descending dysploid *S. tora* karyotype. Chromosomes not involved in a particular process are faded to emphasize chromosome rearrangements events.

In our previous work, we showed that *S. occidentalis* has interstitial telomeric repeats although not as extensive as in *S. tora* (Pellerin et al., 2019). To better understand the chromosomal relationship between *S. occidentalis* and *S. tora*, we performed a comparative FISH between these two species using StoTR02_7_tel and centromeric (StoTR03_178 and StoTR05_180) distribution (Figure 4A).

Both *S. occidentalis* and *S. tora* showed StoTR02_7_tel signals at the canonical sites of chromosome termini. However, aside from being detected in only three chromosomes in *S. occidentalis*, interstitial StoTR02_7_tel sites were much weaker in *S. occidentalis* than in *S. tora*. Moreover, we did not observe any pericentromeric StoTR02_7_tel signals in *S. occidentalis*. Conversely, the interstitial and pericentromeric telomere repeat signals in *S. tora* were more ubiquitous and more pronounced suggesting that these interstitial telomere repeat loci in *S. tora* could stretch several megabases (Figure 4A).

Another striking difference between *S. occidentalis* and *S. tora* is the chromosomal distribution of StoTR05_180. In *S. occidentalis*, StoTR05_180 was cytologically colocalized with StoTR02_7_tel at chromosome termini and at interstitial sites. Moreover, some chromosomes have weaker StoTR05_180 signals than StoTR02_7_tel, indicating fewer copies (Figure 4A). In *S. tora*, terminal StoTR05_180 was not detected, but the interstitial and pericentromeric StoTR05_180 signals were considerably more intense.

## Discussion

We identified eight tandem repeats in the *S. tora* genome through clustering of homologous reads from 1× whole-genome short reads. These repeats covered >12% of the *S. tora* genome. The striking differences in the chromosomal distribution of these repeats between *S. occidentalis* and *S. tora* (i) provides cytological evidence of extensive chromosomal rearrangements that have occurred during *S. tora* speciation, (ii) highlights the roles of tandem repeats in these rearrangements, and (iii) offer a system for identifying individual *S. tora* chromosomes for karyotyping and cytotaxonomic studies.

### Roles of Tandem Repeats in *S. tora* Karyotype Evolution

The numerous ectopic and highly amplified tandem repeats in the (peri)centromeric and interstitial regions in *S. tora* chromosomes is evidence that they have been involved in shaping the extant *S. tora* genome (Figure 4B), either as a cause or, more likely, as a consequence of illegitimate recombination (Murat et al., 2010). This evidence, relative to that of *S. occidentalis* includes: (i) the ectopic loci of interstitial and pericentromeric telomeric repeats sites besides the canonical terminal telomeric sites, (ii) the displacement of StoTR05_180 from subtelomeric in *S. occidentalis* to interstital and pericentromeric sites in *S. tora*; (iii) the amplification of StoTR03_178 specifically in *S. tora*; (iv) the presence of StoTR06_159 homologous sequences in the 45S rDNA IGS; and (v) the descending dysploid karyotype of *S. tora*.

First, interstitial telomeric repeats (ITR) have been observed in several plants, although some are not as extensive as what is seen here in *S. tora* (Fuchs et al., 1995; He et al., 2013). These ITRs may have been generated via descending dysploid mechanisms such as end fusions (EET) and chromosome insertions (NCI), via chromosome inversions, or via amplification and reintegration of telomeric extrachromosomal circular DNA (eccDNA) repeats (Zellinger et al., 2007; Cohen and Segal, 2009). Although the replication, amplification, anchoring, and reintegration of eccDNAs into chromosomes have been demonstrated (Zellinger et al., 2007; Cohen and Segal, 2009; Durkin et al., 2012; Koo et al., 2018), we are careful not to speculate about their supposed contribution to generating interstitial and pericentromeric telomeric repeats in *S. tora* because there is limited information about the mechanisms of eccDNA-induced chromosome rearrangements in plants. Nevertheless, mechanisms involving eccDNA (Cohen and Segal, 2009) could help explain the amplification of ectopic telomeric repeats after they have moved to their new sites.

Alternatively, both EET and NCI could generate ITRs. However, EET is more plausible for explaining the situation in *S. tora* because all interstitial telomeric signals were observed only in one arm of all chromosomes bearing this signal, contrary to both arms if they were caused by NCI. However, we cannot rule out the possibility of NCI-mediated chromosome rearrangements, which, in this case, should require a biased reduction or elimination of the interstitial telomeric repeat array size in one arm, leaving signals in one arm undetectable by FISH (Majerová et al., 2014). Nevertheless, we believe that this is unlikely to happen compared with EET, considering the low likelihood for all chromosomes with interstitial telomeric loci to receive the same single-arm signals in a presumably random process of chromosomal rearrangements.

Second, whereas EET could generate interstitial telomeric and StoTR05_180 loci, it does not explain the disappearance of subtelomeric StoTR05_180 and its subsequent accumulation at pericentromeres as well as the concomitant accumulation of pericentromeric telomeric repeat loci in *S. tora*. Sequence microhomology between telomeres and centromeres (He et al., 2013; Pellestor and Gatinois, 2018) may have caused paracentric inversion with breakpoints at a proximal pericentromeric region and at terminal telomeric repeat loci (rather than at subtelomeric StoTR05_180 loci), which then likely generated pericientromeric StoTR05_180 at recombination sites followed by repeat array amplification. The subsequent disruption of (peri)centromere chromatin could have altered the epigenetic makeup of these regions, promoting centromere repositioning at pericentromeric StoTR05_180 sites, as satellites are known targets for epigenetic *de novo* centromere formation (Okada et al., 2007; Schubert, 2018; Lu and He, 2019), and centromere repositioning is shown to be more frequent than originally thought (Mandáková et al., 2020). Repositioned kinetochore assembly at pericentromeric StoTR05_180 sites likely seeded DNA mutation and amplification of StoTR03_178 as a new centromeric repeat variant unique to *S. tora*.

Third, the fact that there are more abundant copies of StoTR03_178 than there are of StoTR05_180, in addition to its centromeric location, suggest a novel shift of centromeric repeat preference to StoTR03_178 resulting from reestablishing proper meiotic pairing after genomic shock (Ma and Gustafson, 2005; Schubert and Vu, 2016). Mutations in StoTR05_180 sequences likely reduced its affinity to the kinetochore, thus weakening the centromere drive (Schubert, 2018). These mutations could have seeded StoTR03_178, which may have developed a higher affinity for the *S. tora* kinetochore and eventually dominated the centromere, pushing the StoTR05_180 to the pericentromeric regions, similar to what has been observed in other plants (Hirsch and Jiang, 2012). In addition, StoTR03_178 was completely absent in *S. occidentalis*, indicating a more recent amplification in the *S. tora* genome. Immunostaining with *S. tora* CENH3 should provide insights into the active centromere sites in *S. tora* chromosomes.

Fourth, 45S rDNA IGS is often linked to genome rearrangements (Havlová et al., 2016). Several duplicated sequences in the 45S rDNA IGS region have been identified as independent satellites somewhere else in the genome, outside the 45S rDNA array, in several plants similar to the relationship between StoTR06_159 and StoIGS_188 (Almeida et al., 2012; Elliott et al., 2013; Kirov et al., 2018). This observation suggests the role of the 45S rDNA IGS as a “repeat carrier” during genome rearrangement. However, although some authors hypothesized that these repeats are from the IGS region, which then moved out and amplified in other chromosomal loci (Almeida et al., 2012), others hypothesize otherwise (Falquet et al., 1997). To date, there is no definitive conclusion to the directionality of IGS-related satellite evolution nor is there a conclusive mechanism to explain the process by which 45S rDNA IGS operates in this process (Almeida et al., 2012).

Lastly, the 2*n* = 28 chromosome number is predominant in *Senna* and lower numbers such as 2*n* = 22–26 are considered descending dysploid species (Cordeiro and Felix, 2017; Pellerin et al., 2019). In *S. tora*, this reduction is likely caused by a merger of two chromosomes, resulting in derivative chromosome 7, as indicated by the interstitial signal of StoTR03_178 in chromosome 7L (Figure 3). The reduced chromosome number in *S. tora* indicates extensive genome rearrangements in *S. tora* relative to that of *S. occidentalis* (Figure 4B).

Chromosome rearrangements in many species often involve just one or a few chromosomes, contrary to all chromosomes rearranging all at once in a concerted manner (Mandáková and Lysak, 2018). However, chromoanagenic pathways, such as chromothripsis and chromoplexy, have been shown to produce massive chromosome rearrangements involving several chromosomes via dysploid and non-dysploid mechanisms in just a few generations (Comai and Tan, 2019; Pellestor and Gatinois, 2020). It is therefore interesting to know whether the extensive chromosomal rearrangements in *S. tora* occurred in a rapid process, or over slow recurrent rounds of hybridization. In addition, considering that *S. occidentalis* has three chromosomes with interstitial StoTR02_7_tel and StoTR05_180, reconstructing the ancestral *Senna* karyotype will be necessary to understand karyotype evolution in *Senna*. Comprehensive comparative cytogenomic analyses in *Senna* are crucial to achieving this.

### A Hypothetical Chromosomal Evolution in *S. tora*

WGD is pervasive in angiosperms (Jiao et al., 2011; Soltis and Soltis, 2016). During the diploidization process, which a cell's mechanism for reestablishing proper meiotic pairing after a genomic shock from WGD (Ma and Gustafson, 2005; Schubert and Vu, 2016), karyotype dysploidy may occur (Levin, 2020). Recurrent cycles of WGD and diploidization in some lineages increased the chances of genome rearrangements and dysploidy, producing taxa with different chromosome numbers (Lysak et al., 2006; Mandakova et al., 2010; Mandáková et al., 2019; Symonds et al., 2010; Jiao et al., 2011; Chalhoub et al., 2014; Murat et al., 2017; Mandáková and Lysak, 2018).

In *Senna, x* = 7 is considered as the base chromosome number after the discovery of *Senna rugosa* cytotypes with a haploid chromosome number of *n* = 7 (Resende et al., 2014), suggesting that species with the predominant 2*n* = 28 could technically be diploidized tetraploids. Comparing the chromosomal distribution of *S. tora* tandem repeats between *S. occidentalis* and *S. tora*, we hypothesize that the extant *S. tora* genome, like many angiosperms, has experienced at least two rounds of WGD in the immediate past. The diploidization that followed each WGD event may have differently influenced the *S. tora* karyotype temporally; such that, the former WGD may have radically reverted the chromosome number to the diploid count whereas the latter “fused” two chromosomes, generating the extant 2*n* = 26 dysploid karyotype (Figure 4B).

After an older WGD event, the chromosome number may have doubled to 2*n* = 28 from an ancestral 2*n* = 14 karyotype. Dysploidy and non-dysploidy mechanisms may have reverted the chromosome number to 2*n* = 14; a similar reversion of chromosomal number to diploid count has been observed between *Zea mays* and *Sorghum bicolor* (Murat et al., 2010; Freeling et al., 2012). A more recent WGD event may have doubled the chromosome number to 2*n* = 28, which is then followed by EET of two chromosomes generating chromosome 7; hence, the extant 2*n* = 26 karyotype of *S. tora*.

Nevertheless, with purely molecular cytogenetic data, it is difficult to draw a definitive conclusion on the chromosome evolution of *S. tora*. The current genome assembly of *S. tora* and comparative genome analyses in *Senna* should provide more insight into *S. tora* chromosome evolution.

### Tandem Repeats for *Senna* Karyotyping and Cytogenetics

The chromosomal distribution of the major *S. tora* tandem repeats facilitated the identification of individual homologous chromosomes for karyotyping (Figure 3B, Supplementary Table 2). Because chromosomal rearrangements are less likely homoplastic, they can be used to infer phylogenetic relationships between species (Mandáková and Lysak, 2008). Repeats are used as species identifiers in cytotaxonomic studies (Guerra, 2008, 2012) because certain repeat families are widely distributed within a taxonomic family or genus, or even specific to a species, a genome, a tissue, or even a chromosome (Sharma and Raina, 2005; Ruban et al., 2014).

To gain a comprehensive understanding of the satellite repeat dynamics in *Senna*, a comprehensive comparative satellitome (Ruiz-Ruano et al., 2016) between *Senna* species will unravel clade- or species-specific abundant satellites in *Senna* not only for karyotyping but also for unraveling satellite dynamics and genome history in *Senna*.

## Conclusion

The identification and chromosomal mapping of the major tandem repeats in *S. tora* provided cytological evidence of past genome rearrangements. We have presented here the major tandem repeats that comprise and play an active role in shaping the highly rearranged descending dysploid *S. tora* karyotype. Using *in silico* and FISH data, we hypothesized an evolutionary pathway to the extant *S. tora* genome involving dysploid and non-dysploid mechanisms. This chromosomal information should be complemented with molecular data because sequences used here were purely generated *in silico*. Amplification and sequencing of the >4 kb *S. tora* 45S rDNA IGS region should provide a more accurate description of the IGS structure, for example, discovery of phylogenetically important sequence variants that would help decipher not only subgenome relationships within *S. tora* but also with other *Senna* species. Moreover, comparison with different *Senna* species should provide further insights into the role of 45S rDNA in genome rearrangements. Lastly, phylogenomic analysis will allow testing for a correlation between chromosomal rearrangements and species divergence and trace major chromosomal events that have occurred during speciation in *Senna*.

To understand whether the repeats identified here are specific to *S. tora* or are conserved within the genus or its sub-lineages, comparative cytogenetic analyses with other related species are necessary. Moreover, whereas most repeats identified here are satellite DNAs, a comprehensive repeatomics (Macas et al., 2015) involving repeats like transposable elements in *S. tora* and other *Senna* species will be the focus of a future work to fully understand the repeat dynamics in *Senna*. The release of the *S. tora* genome should enable identification of the two donor chromosomes that contribute to the *S. tora* chromosome 7 and pave the way for reconstructing the ancestral *Senna* karyotype.

## Data Availability Statement

The datasets presented in this study can be found in online repositories. The names of the repository/repositories and accession number(s) can be found in the article/Supplementary Material.

## Author Contributions

NW and HK conceived and designed the experiment. NW performed bioinformatics analyses. RP performed the FISH experiment. NW and RP wrote the manuscript. S-HK provided the whole genome sequence and plant samples. All authors approved the final manuscript.

## Conflict of Interest

The authors declare that the research was conducted in the absence of any commercial or financial relationships that could be construed as a potential conflict of interest.

## References

[B1] Almeida C. Fonseca A. Dos Santos K. G. Mosiolek M. Pedrosa-Harand A. (2012). Contrasting evolution of a satellite DNA and its ancestral IGS rDNA in Phaseolus (Fabaceae). Genome 55, 683–689. 10.1139/g2012-059 23050694

[B2] Altschul S. F. Gish W. Miller W. Myers E. W. Lipman D. J. (1990). Basic local alignment search tool. J. Mol. Biol. 215, 403–410. 10.1016/S0022-2836(05)80360-2 2231712

[B3] Benson G. (1999). Tandem repeats finder: a program to analyze DNA sequences. Nucleic Acids Res. 27, 573–580. 10.1093/nar/27.2.573 9862982 PMC148217

[B4] Chalhoub B. Denoeud F. Liu S. Parkin I. A. P. Tang H. Wang X.. (2014). Early allopolyploid evolution in the post-Neolithic Brassica napus oilseed genome. Science 345, 950–953. 10.1126/science.1253435 25146293

[B5] Charlesworth B. Sniegowski P. Stephan W. (1994). The evolutionary dynamics of repetitive DNA in eukaryotes. Nature 371, 215–220. 10.1038/371215a0 8078581

[B6] Chung K. S. Weber J. A. Hipp A. L. (2011). Dynamics of chromosome number and genome size variation in a cytogenetically variable sedge (Carex scoparia var. scoparia, Cyperaceae). Am. J. Bot. 98, 122–129. 10.3732/ajb.1000046 21613090

[B7] Cohen S. Segal D. (2009). Extrachromosomal circular DNA in eukaryotes: possible involvement in the plasticity of tandem repeats. Cytogenet. Genome Res. 124, 327–338. 10.1159/000218136 19556784

[B8] Comai L. Tan E. H. (2019). Haploid induction and genome instability. Trends Genet. 35, 791–803. 10.1016/j.tig.2019.07.005 31421911

[B9] Cordeiro J. M. P. Felix L. P. (2017). Intra-and interspecific karyotypic variations of the genus *Senna Mill* (Fabaceae, Caesalpinioideae). Acta Botanica Brasilica 32, 128–134. 10.1590/0102-33062017abb0274

[B10] Durkin K. Coppieters W. Drögemüller C. Ahariz N. Cambisano N. Druet T.. (2012). Serial translocation by means of circular intermediates underlies colour sidedness in cattle. Nature 482, 81–84. 10.1038/nature10757 22297974

[B11] Elliott T. A. Stage D. E. Crease T. J. Eickbush T. H. (2013). In and out of the rRNA genes: characterization of Pokey elements in the sequenced Daphnia genome. Mob. DNA 4:20. 10.1186/1759-8753-4-20 24059783 PMC3849761

[B12] Falquet J. Creusot F. Dron M. (1997). Molecular analysis of *Phaseolus vulgaris* rDNA unit and characterization of a satellite DNA homologous to IGS subrepeats. Plant Physiol. Bioch. 35, 611–622.

[B13] Fedoroff N. V. (2012). Presidential address. Transposable elements, epigenetics, and genome evolution. Science 338, 758–767. 10.1126/science.338.6108.758 23145453

[B14] Fedoroff N. V. Bennetzen J. L. (2013). “Transposons, genomic shock, and genome evolution,” in Plant Transposons and Genome Dynamics in Evolution, ed N. V. Fedoroff (Hoboken, NJ: Wiley-Blackwell), 181–201. 10.1002/9781118500156.ch10

[B15] Freeling M. Woodhouse M. R. Subramaniam S. Turco G. Lisch D. Schnable J. C. (2012). Fractionation mutagenesis and similar consequences of mechanisms removing dispensable or less-expressed DNA in plants. Curr. Opin. Plant Biol. 15, 131–139. 10.1016/j.pbi.2012.01.015 22341793

[B16] Frello S. Heslop-Harrison J. S. (2000). Repetitive DNA sequences in Crocus vernus Hill (Iridaceae): The genomic organization and distribution of dispersed elements in the genus Crocus and its allies. Genome 43, 902–909. 10.1139/g00-044 11081982

[B17] Fuchs J. Brandes A. Schubert I. (1995). Telomere sequence localization and karyotype evolution in higher plants. Plant Syst. Evol. 196, 227–241. 10.1007/BF00982962

[B18] Garrido-Ramos M. (2017). Satellite DNA: an evolving topic. Genes 8:230 10.3390/genes8090230 PMC5615363 28926993

[B19] Garrido-Ramos M. A. (2015). Satellite DNA in plants: more than just rubbish. Cytogenet. Genome Res. 146, 153–170. 10.1159/000437008 26202574

[B20] Guerra M. (2008). Chromosome numbers in plant cytotaxonomy: concepts and implications. Cytogenet. Genome Res. 120, 339–350. 10.1159/000121083 18504363

[B21] Guerra M. (2012). Cytotaxonomy: the end of childhood. Plant Biosyst. 146, 703–710. 10.1080/11263504.2012.717973

[B22] Hartley G. O'Neill R. (2019). Centromere repeats: hidden gems of the genome. Genes 10:223. 10.3390/genes10030223 30884847 PMC6471113

[B23] Havlová K. Dvoráčková M. Peiro R. Abia D. Mozgová I. Vansáčová L.. (2016). Variation of 45S rDNA intergenic spacers in *Arabidopsis thaliana*. Plant Mol. Biol. 92, 457–471. 10.1007/s11103-016-0524-1 27531496

[B24] He L. Liu J. Torres G. A. Zhang H. Jiang J. Xie C. (2013). Interstitial telomeric repeats are enriched in the centromeres of chromosomes in Solanum species. Chromosome Res. 21, 5–13. 10.1007/s10577-012-9332-x 23250588

[B25] Hirsch C. D. Jiang J. (2012). “Centromeres: sequences, structure, and biology plant genome diversity,” in Plant Genome Diversity, eds Wendel J. F. Greilhuber J. Dolezel J. Leitch I. J. (Vienna: Springer), 59–70.

[B26] Ijdo J. W. Baldini A. Ward D. C. Reeders S. T. Wells R. A. (1991). Origin of human chromosome 2: an ancestral telomere-telomere fusion. PNAS 88, 9051–9055. 10.1073/pnas.88.20.9051 1924367 PMC52649

[B27] Imai H. T. Taylor R. W. (1989). Chromosomal polymorphisms involving telomere fusion, centromeric inactivation and centromere shift in the ant Myrmecia (pilosula) n=1. Chromosoma 98, 456–460. 10.1007/BF00292792

[B28] Jang D. S. Lee G. Y. Kim Y. S. Lee Y. M. Kim C.-S. Yoo J. L.. (2007). Anthraquinones from the seeds of *Cassia tora* with inhibitory activity on protein glycation and aldose reductase. Biol. Pharm. Bull. 30, 2207–2210. 10.1248/bpb.30.2207 17978503

[B29] Jiao Y. Wickett N. J. Ayyampalayam S. Chanderbali A. S. Landherr L. Ralph P. E. (2011). Ancestral polyploidy in seed plants and angiosperms. Nature 473, 97–100. 10.1038/nature09916 21478875

[B30] Kang S.-H. Pandey R. P. Lee C.-M. Sim J.-S. Jeong J.-T. Choi B.-S.. (2020a). Genome-enabled discovery of anthraquinone biosynthesis in *Senna tora*. Nat. Commun. 2020:063495. 10.1101/2020.04.27.063495 33208749 PMC7674472

[B31] Kang S. H. Lee W. H. Lee C. M. Sim J. S. Won S. Y. Han S. R.. (2020b). *De novo* transcriptome sequence of *Senna tora* provides insights into anthraquinone biosynthesis. PLoS ONE 15:e0225564. 10.1371/journal.pone.0225564 32380515 PMC7205477

[B32] Kasinathan S. Henikoff S. (2018). Non-B-form DNA is enriched at centromeres. Mol. Biol. Evol. 35, 949–962. 10.1093/molbev/msy010 29365169 PMC5889037

[B33] Kim K. Lee S.-C. Lee J. Yu Y. Yang K. Choi B.-S.. (2015). Complete chloroplast and ribosomal sequences for 30 accessions elucidate evolution of Oryza AA genome species. Sci. Rep. 5:15655. 10.1038/srep15655 26506948 PMC4623524

[B34] Kirov I. Gilyok M. Knyazev A. Fesenko I. (2018). Pilot satellitome analysis of the model plant, *Physcomitrella patens*, revealed a transcribed and high-copy IGS related tandem repeat. Comp. Cytogenet. 12, 493–513. 10.3897/CompCytogen.v12i4.31015 30588288 PMC6302065

[B35] Koo D.-H. Molin W. T. Saski C. A. Jiang J. Putta K. Jugulam M.. (2018). Extrachromosomal circular DNA-based amplification and transmission of herbicide resistance in crop weed *Amaranthus palmeri*. PNAS 115, 3332–3337. 10.1073/pnas.1719354115 29531028 PMC5879691

[B36] Kubis S. Schmidt T. Heslop-Harrison J. S. (1998). Repetitive DNA elements as a major component of plant genomes. Ann. Bot. 82, 45–55. 10.1006/anbo.1998.0779

[B37] Kursel L. E. Malik H. S. (2016). Centromeres. Curr. Biol. 26, R487–R490. 10.1016/j.cub.2016.05.031 27326706

[B38] Levin D. A. (2020). Did dysploid waves follow the pulses of whole genome duplications? Plant Syst. Evol. 306:75 10.1007/s00606-020-01704-5

[B39] Lim K. B. Yang T. J. Hwang Y. J. Kim J. S. Park J. Y. Kwon S. J.. (2007). Characterization of the centromere and peri-centromere retrotransposons in Brassica rapa and their distribution in related Brassica species. Plant J. 49, 173–183. 10.1111/j.1365-313X.2006.02952.x 17156411

[B40] Lim K. Y. Skalicka K. Koukalova B. Volkov R. A. Matyasek R. Hemleben V.. (2004). Dynamic changes in the distribution of a satellite homologous to intergenic 26-18S rDNA spacer in the evolution of Nicotiana. Genetics 166, 1935–1946. 10.1534/genetics.166.4.1935 15126410 PMC1470824

[B41] Long Q. Rabanal F. A. Meng D. Huber C. D. Farlow A. Platzer A.. (2013). Massive genomic variation and strong selection in *Arabidopsis thaliana* lines from Sweden. Nature Genet. 45, 884–890. 10.1038/ng.2678 23793030 PMC3755268

[B42] Louzada S. Lopes M. Ferreira D. Adega F. Escudeiro A. Gama-Carvalho M.. (2020). Decoding the role of satellite DNA in genome architecture and plasticity-an evolutionary and clinical affair. Genes 11:72. 10.3390/genes11010072 31936645 PMC7017282

[B43] Lu M. He X. (2019). Centromere repositioning causes inversion of meiosis and generates a reproductive barrier. Proc. Natl. Acad. Sci. U. S. A. 116, 21580–21591. 10.1073/pnas.1911745116 31597736 PMC6815110

[B44] Luo M. C. Deal K. R. Akhunov E. D. Akhunova A. R. Anderson O. D. Anderson J. A.. (2009). Genome comparisons reveal a dominant mechanism of chromosome number reduction in grasses and accelerated genome evolution in Triticeae. PNAS 106, 15780–15785. 10.1073/pnas.0908195106 19717446 PMC2747195

[B45] Lysak M. A. Berr A. Pecinka A. Schmidt R. McBreen K. Schubert I. (2006). Mechanisms of chromosome number reduction in Arabidopsis thaliana and related Brassicaceae species. PNAS 103, 5224–5229. 10.1073/pnas.0510791103 16549785 PMC1458822

[B46] Ma X. F. Gustafson J. (2005). Genome evolution of allopolyploids: a process of cytological and genetic diploidization. Cytogenet. Genome Res. 109, 236–249. 10.1159/000082406 15753583

[B47] Macas J. Novak P. Pellicer J. Cizkova J. Koblizkova A. Neumann P.. (2015). In depth characterization of repetitive DNA in 23 plant genomes reveals sources of genome size variation in the legume tribe Fabeae. PLoS ONE 10:e0143424. 10.1371/journal.pone.0143424 26606051 PMC4659654

[B48] Majerová E. Mandáková T. Vu G. T. H. Fajkus J. Lysak M. A. Fojtová M. (2014). Chromatin features of plant telomeric sequences at terminal vs. internal positions. Front. Plant Sci. 5:593. 10.3389/fpls.2014.00593 25408695 PMC4219495

[B49] Mandáková T. Hloušková P. Koch M. A. Lysak M. A. (2020). Genome evolution in Arabideae was marked by frequent centromere Repositioning 32, 650–665. 10.1105/tpc.19.00557 PMC7054033 31919297

[B50] Mandakova T. Joly S. Krzywinski M. Mummenhoff K. Lysak M. A. (2010). Fast diploidization in close mesopolyploid relatives of Arabidopsis. Plant Cell 22, 2277–2290. 10.1105/tpc.110.074526 20639445 PMC2929090

[B51] Mandáková T. Lysak M. A. (2008). Chromosomal phylogeny and karyotype evolution in x=7 *Crucifer* species (Brassicaceae). Plant Cell 20, 2559–2570. 10.1105/tpc.108.062166 18836039 PMC2590746

[B52] Mandáková T. Lysak M. A. (2018). Post-polyploid diploidization and diversification through dysploid changes. Curr. Opin. Plant Biol. 42, 55–65. 10.1016/j.pbi.2018.03.001 29567623

[B53] Mandáková T. Pouch M. Brock J. R. Al-Shehbaz I. A. Lysak M. A. (2019). Origin and evolution of diploid and allopolyploid camelina genomes were accompanied by chromosome shattering. Plant Cell 31, 2596–2612. 10.1105/tpc.19.00366 31451448 PMC6881126

[B54] Mandáková T. Pouch M. Harmanová K. Zhan S. H. Mayrose I. Lysak M. A. (2017). Multispeed genome diploidization and diversification after an ancient allopolyploidization. Mol. Ecol. 26, 6445–6462. 10.1111/mec.14379 29024107

[B55] Marazzi B. Endress P. K. Queiroz L. P. Conti E. (2006). Phylogenetic relationships within Senna (Leguminosae, Cassiinae) based on three chloroplast DNA regions: patterns in the evolution of floral symmetry and extrafloral nectaries. Am. J. Bot. 93, 288–303. 10.3732/ajb.93.2.288 21646190

[B56] Mehrotra S. Goyal V. (2014). repetitive sequences in plant nuclear dna: types, distribution, evolution and function. Genom. Proteom. Bioinform. 12, 164–171. 10.1016/j.gpb.2014.07.003 25132181 PMC4411372

[B57] Murat F. Armero A. Pont C. Klopp C. Salse J. (2017). Reconstructing the genome of the most recent common ancestor of flowering plants. Nature Genet. 49, 490–496. 10.1038/ng.3813 28288112

[B58] Murat F. Xu J.-H. Tannier E. Abrouk M. Guilhot N. Pont C.. (2010). Ancestral grass karyotype reconstruction unravels new mechanisms of genome shuffling as a source of plant Evolution 20, 1545–1557. 10.1101/gr.109744.110 20876790 PMC2963818

[B59] Novák P. Robledillo L. A. Koblizková A. Vrbová I. Neumann P. Macas J. (2017). TAREAN: a computational tool for identification and characterization of satellite DNA from unassembled short reads. Nucleic Acids Res. 45:e111. 10.1093/nar/gkx257 28402514 PMC5499541

[B60] Ohri D. Kumar A. Pal M. (1986). Correlations between 2C DNA values and habit inCassia (Leguminosae:Caesalpinioideae). Plant Syst. Evol. 153, 223–227. 10.1007/BF00983689

[B61] Okada T. Ohzeki J.-i. Nakano M. Yoda K. Brinkley W. R. Larionov V.. (2007). CENP-B controls centromere formation depending on the chromatin context. Cell 131, 1287–1300. 10.1016/j.cell.2007.10.045 18160038

[B62] Pellerin R. Waminal N. Kim H. (2019). FISH mapping of rDNA and telomeric repeats in 10 *Senna* species. Hortic. Environ. Biotechnol. 60, 253–260. 10.1007/s13580-018-0115-y

[B63] Pellestor F. Gatinois V. (2018). Chromoanasynthesis: another way for the formation of complex chromosomal abnormalities in human reproduction. Hum. Reprod. 33, 1381–1387. 10.1093/humrep/dey231 30325427

[B64] Pellestor F. Gatinois V. (2020). Chromoanagenesis: a piece of the macroevolution scenario. Mol. Cytogenet. 13:3. 10.1186/s13039-020-0470-0 32010222 PMC6988253

[B65] Perumal S. Waminal N. E. Lee J. Lee J. Choi B. S. Kim H. H.. (2017). Elucidating the major hidden genomic components of the A, C, and AC genomes and their influence on *Brassica* evolution. Sci. Rep. 7:17986. 10.1038/s41598-017-18048-9 29269833 PMC5740159

[B66] Puri B. K. (2018). The potential medicinal uses of Cassia tora linn leaf and seed extracts. Rev. Recent Clin. Trials 13, 3–4. 10.2174/157488711301180131145359 29469686

[B67] Resende K. Prado C. Davide L. Torres G. J. T. J. o. B. (2014). Polyploidy and apomixis in accessions of *Senna rugosa* (G. Don). HS Irwin Barneby 38, 510–515. 10.3906/biy-1312-66

[B68] Rosato M. Álvarez I. Feliner G. N. Rosselló J. A. (2018). Inter- and intraspecific hypervariability in interstitial telomeric-like repeats (TTTAGGG)n in Anacyclus (Asteraceae). Ann. Bot. 122, 387–395. 10.1093/aob/mcy079 29800070 PMC6110349

[B69] Rousselet J. Monti L. Auger-Rozenberg M. A. Parker J. S. Lemeunier F. (2000). Chromosome fission associated with growth of ribosomal DNA in Neodiprion abietis (Hymenoptera: Diprionidae). Proceed. Biol. Sci. 267, 1819–1823. 10.1098/rspb.2000.1216 11052531 PMC1690759

[B70] Ruban A. Fuchs J. Marques A. Schubert V. Soloviev A. Raskina O.. (2014). B chromosomes of *Aegilops speltoides* are enriched in organelle genome-derived sequences. PLoS ONE 9:e90214. 10.1371/journal.pone.0090214 24587288 PMC3936023

[B71] Ruiz-Ruano F. J. López-León M. D. Cabrero J. Camacho J. P. M. (2016). High-throughput analysis of the satellitome illuminates satellite DNA evolution. Sci. Rep. 6:28333. 10.1038/srep28333 27385065 PMC4935994

[B72] Salser W. Bowen S. Browne D. el-Adli F. Fedoroff N. Fry K.. (1976). Investigation of the organization of mammalian chromosomes at the DNA sequence level. Fed. Proc. 35, 23–35. 1107072

[B73] Schubert I. (2018). What is behind “centromere repositioning”? Chromosoma 127, 229–234. 10.1007/s00412-018-0672-y 29705818

[B74] Schubert I. Lysak M. A. (2011). Interpretation of karyotype evolution should consider chromosome structural constraints. Trends Genetics 27, 207–216. 10.1016/j.tig.2011.03.004 21592609

[B75] Schubert I. Vu G. T. H. (2016). Genome stability and evolution: attempting a holistic view. Trends Plant Sci. 21, 749–757. 10.1016/j.tplants.2016.06.003 27427334

[B76] Schubert V. Neumann P. Marques A. Heckmann S. Macas J. Pedrosa-Harand A.. (2020). Super-resolution microscopy reveals diversity of plant centromere architecture. Int. J. Mol. Sci. 21:3488. 10.3390/ijms21103488 32429054 PMC7278974

[B77] Sharma S. Raina S. N. (2005). Organization and evolution of highly repeated satellite DNA sequences in plant chromosomes. Cytogenet. Genome Res. 109, 15–26. 10.1159/000082377 15753554

[B78] Shatskikh A. S. Kotov A. A. Adashev V. E. Bazylev S. S. Olenina L. V. (2020). Functional significance of satellite DNAs: insights from *Drosophila*. 8:312. 10.3389/fcell.2020.00312 32432114 PMC7214746

[B79] Soltis P. S. Soltis D. E. (2016). Ancient WGD events as drivers of key innovations in angiosperms. Curr. Opin. Plant Biol. 30, 159–165. 10.1016/j.pbi.2016.03.015 27064530

[B80] Sousa A. Renner S. S. (2015). Interstitial telomere-like repeats in the monocot family Araceae. Bot. J. Linn. Soc. 177, 15–26. 10.1111/boj.12231

[B81] Symonds V. V. Soltis P. S. Soltis D. E. (2010). Dynamics of polyploid formation in Tragopogon (Asteraceae): recurrent formation, gene flow, and population structure. Evolution 64, 1984–2003. 10.1111/j.1558-5646.2010.00978.x 20199558

[B82] Szymanski M. Zielezinski A. Barciszewski J. Erdmann V. A. Karlowski W. M. (2016). 5SRNAdb: an information resource for 5S ribosomal RNAs. Nucleic Acids Res. 44, D180–D183. 10.1093/nar/gkv1081 26490961 PMC4702797

[B83] Vrana J. Simkova H. Kubalakova M. Cihalikova J. Dolezel J. (2012). Flow cytometric chromosome sorting in plants: the next generation. Methods 57, 331–337. 10.1016/j.ymeth.2012.03.006 22440520

[B84] Waminal N. Park H. M. Ryu K. B. Kim J. H. Yang T. J. Kim H. H. (2012). Karyotype analysis of Panax ginseng C.A.Meyer, 1843 (Araliaceae) based on rDNA loci and DAPI band distribution. Comp. Cytogenet. 6, 425–441. 10.3897/CompCytogen.v6i4.3740 24260682 PMC3834566

[B85] Waminal N. E. Pellerin R. J. Kim N.-S. Jayakodi M. Park J. Y. Yang T.-J.. (2018a). Rapid and efficient FISH using pre-labeled oligomer probes. Sci. Rep. 8:8224. 10.1038/s41598-018-26667-z 29844509 PMC5974128

[B86] Waminal N. E. Perumal S. Lee J. Kim H. H. Yang T.-J. (2016). Repeat evolution in Brassica rapa (AA), B. oleracea (CC), and B. napus (AACC) genomes. Plant Breed. Biotech. 4, 107–122. 10.9787/PBB.2016.4.2.107

[B87] Waminal N. E. Perumal S. Liu S. Chalhoub B. Kim H. H. Yang T.-J. (2018b). “Quantity, distribution, and evolution of major repeats in *Brassica napus*,” in The Brassica napus Genome, eds Liu S. Snowdon R. Chalhoub B. (Cham: Springer International Publishing), 111–129. 10.1007/978-3-319-43694-4_6

[B88] Waminal N. E. Yang T.-J. In J.-G. Kim H. H. (2020). Five-color fluorescence in situ hybridization system for karyotyping of Panax ginseng. Horticult. Environ. Biotechnol. 61, 869–877. 10.1007/s13580-020-00267-1

[B89] Wicker T. Sabot F. Hua-Van A. Bennetzen J. L. Capy P. Chalhoub B.. (2007). A unified classification system for eukaryotic transposable elements. Nat. Rev. Genet. 8, 973–982. 10.1038/nrg2165 17984973

[B90] Zellinger B. Akimcheva S. Puizina J. Schirato M. Riha K. (2007). Ku suppresses formation of telomeric circles and alternative telomere lengthening in *Arabidopsis*. Mol. Cell 27, 163–169. 10.1016/j.molcel.2007.05.025 17612498

