# Supplementary material for: Chromosomal Mapping of Tandem Repeats Revealed Massive Chromosomal Rearrangements and Insights Into Senna tora Dysploidy
Source: Front Plant Sci. 2021 Feb 10;12:629898. doi: 10.3389/fpls.2021.629898 (PMC7902697; doi:10.3389/fpls.2021.629898)
Supplement: Supplementary file 1 [file Data_Sheet_1.docx]

*Supplementary Material*

**Suppementary Figures**


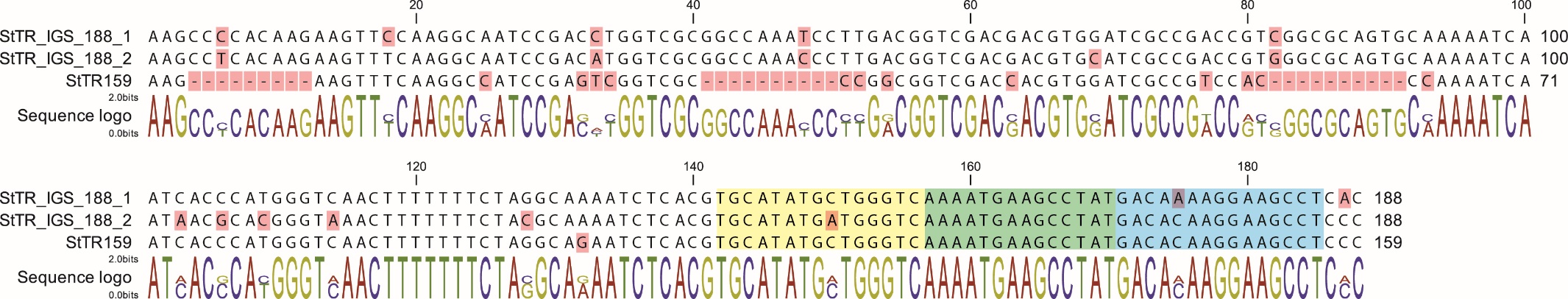


**Supplementary Figure 1.** Sequence alignment of StoIGSTR_188 and StoTR06_159. Two complete StoIGSTR_188 from the 45S rDNA IGS region and the StTR159 consensus are shown. A total of 29 bp from three regions (nt 4–12, 41–50, and 82–91) were deleted in StTR159. The FISH targets of StoTR06_159_OP1 and StoTR06_159_OP2 are shown in yellow and blue highlights, respectively. The green highlight shows the overlapping regions between targets of StoTR06_159_OP1 and StoTR06_159_OP2 but because each probe is strand-specific, they do not compete for target DNA.


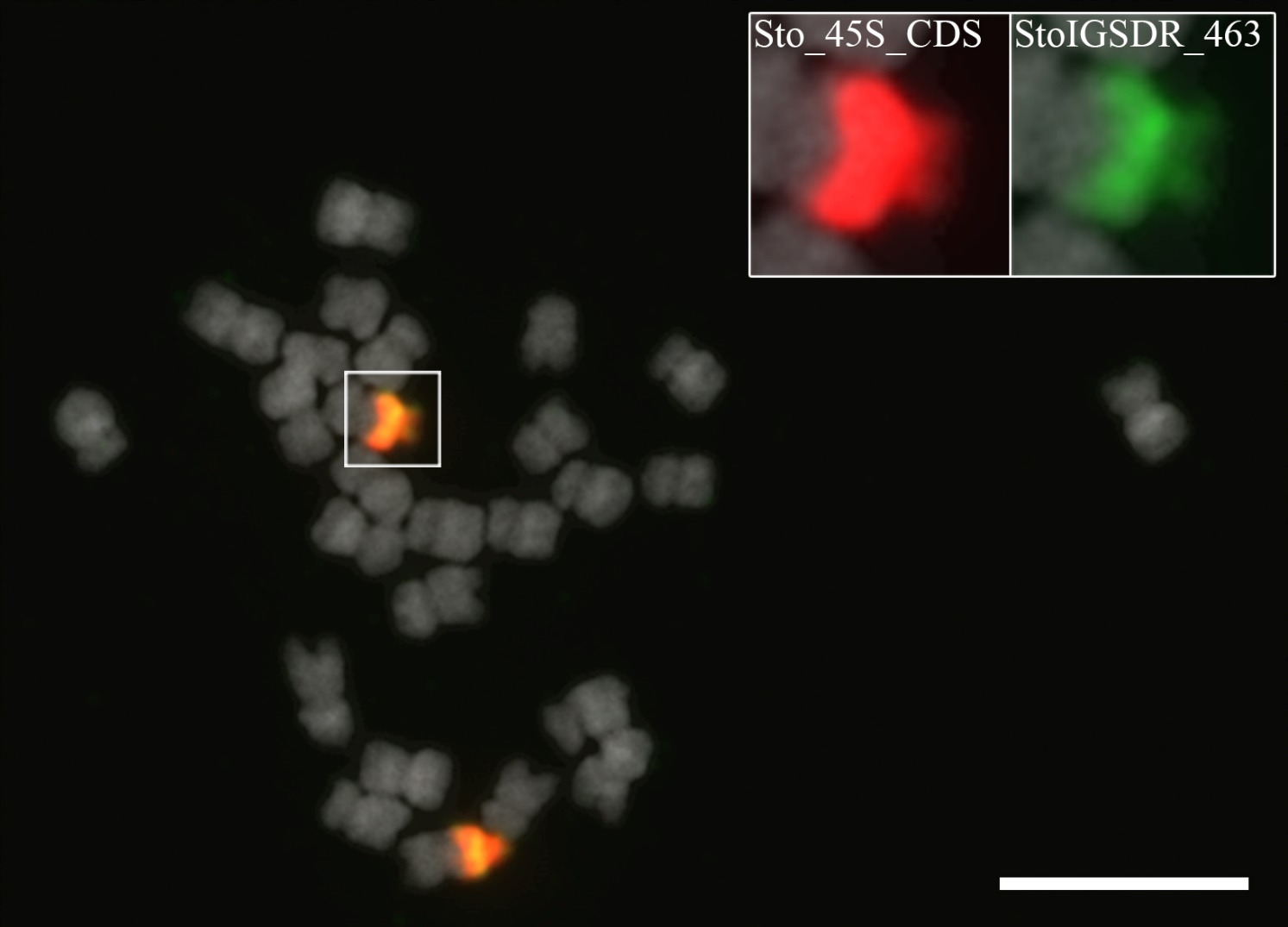


**Supplementary Figure 2.** FISH of 45S rDNA coding sequence and St_IGS_DR463. PLOPs from 18S rDNA and St_IGS_DR463 colocalized at the NOR site in chromosome 3S. No extra-NOR signal was observed for St_IGS_DR463. Bar = 10 µm.


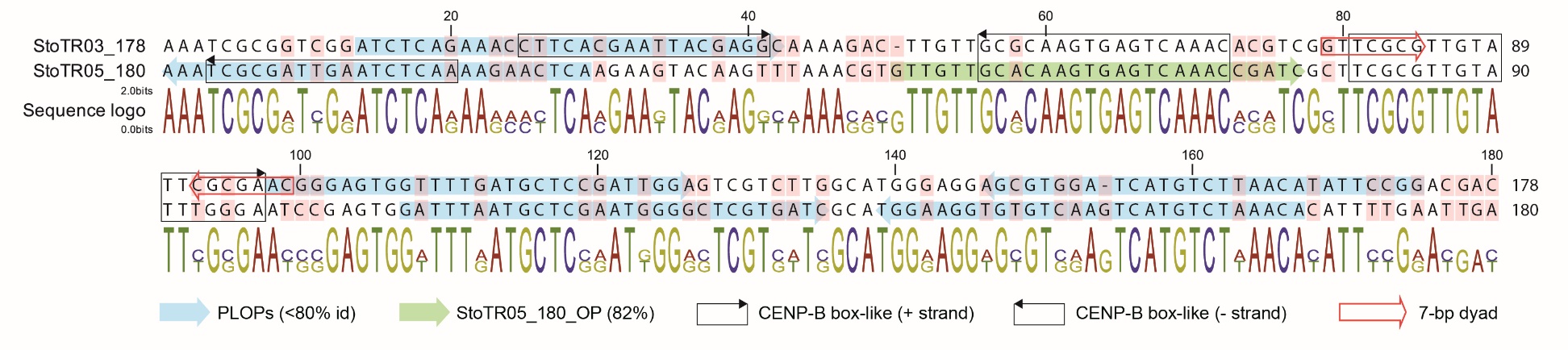


**Supplementary Figure 3.** Sequence alignment of StoTR03_178 and StoTR05_180. A) Sequence alignment of StoTR03_178 and StoTR05_180 consensus sequences showed 71%. Highlighted nucleotides are polymorphic sites. Colored background arrows are target regions of PLOPs listed in Supplementary Table 1: blue for < 80% identity with non-target sequence and green for StoTR05_180_OP2 which has > 82%. Boxed sequences correspond to CENP-B box-like sequences. Boxed red arrows show the 7 bp palindromic dyad symmetry unique to StoTR03_178.

**Supplementary Table 1.** List of the primers and PLOPs used for FISH^a^

| **Name** | **Oligo Name** | **Primer/PLOP sequences (5′—3′)** | **Length**  **(bp)** | **Site in consensus** | **Modification** |
| --- | --- | --- | --- | --- | --- |
| StoTR01_86 | StoTR01_86_OP1 | TTAATCAGTTTTCGCCGATGAGTGTTTCG | 29 | 44..72 | 5′-FAM |
|  | StoTR01_86_OP2 | CATCAGTTTTCGCCAATGAGTGTTTCG | 27 | 4..30 |  |
| StoTR03_178 | StoTR03_178_OP1 | CCGGAATATGTTAAGACATGATCCACGCT | 29 | 145..173 | 5′-Cy5 |
|  | StoTR03_178_OP2 | ATCTCAGAAACCTTCACGAATTACGAGGC | 29 | 14..42 |  |
|  | StoTR03_178_OP3 | CCGGAGTGGTTTTGATGCTCCAATTGGA | 28 | 98..125 |  |
| StoTR04_55 | StoTR04_55_OP | GCGAAAACTGATTAAAAAAAGAAAAATGAATATCAAG | 37 | 2..38 | 5′-AMCA |
| StoTR05_180 | StoTR05_180_OP1 | GATTTAATGCTCGAATGGGGCTCGTGATC | 29 | 62..90 | 5′-Texas Red |
|  | StoTR05_180_OP2 | GTTGTTGCACAAGTGAGTCAAACCGATC | 28 | 5..32 |  |
|  | StoTR05_180_OP3 | TGTTTAGACATGACTTGACACACCTTCCA | 29 | 94..122 |  |
|  | StoTR05_180_OP4 | TGAGTTCTTTTGAGATTCAATCGCGATTT | 29 | 136..164 |  |
| StoTR06_159 | StoTR06_159_OP1 | TGCATATGCTGGGTCAAAATGAAGCCTAT | 29 | 36..64 | 5′-Cy3 |
|  | StoTR06_159_OP2 | AGGCTTCCTTGTGTCATAGGCTTCATTTT | 29 | 21..49 |  |
| StoIGS_463 | StoIGS_463_PLOP1 | AAACCAATATATATTCTATTTTTCGTGATT | 30 | 17..46 | 5′-FAM |
|  | StoIGS_463_PLOP2 | CAAATGATTGATAAGCCTTTAATTTTATTA | 30 | 60..89 |  |
|  | StoIGS_463_PLOP3 | GAAATTTTGGGGTTAAGCTTATATATTTTT | 30 | 256..258 |  |

^a^For 45S rDNA, 5S rDNA, and telomere repeats, see Waminal et al. (2018).

**Supplementary Table 2.** Summary of distribution of the *Senna* repeats in the 13 *Senna tora* chromosomes.

| **Chr. No.** | **Chromosome features** |
| --- | --- |
| 1 | Intercalary colocalization of the the StoTR02_7_tel and StoTR04_55 on the long arm (L); paracentromeric colocalization of the DAPI bands, StoTR04_55 and StoTR01_86 on small arm (S); centromeric colocalization of the the StoTR02_7_tel and all StTRs except StoTR01_86, the StoTR02_7_tel on the both termini regions. |
| 2 | Sto_45S_CDS colocalization of the major signals of StoTR06_159 on NOR; the intercalary colocalization of StoTR04_55, StoTR06_159 on 2S; paracentromeric colocalization of the StoTR02_7_tel, StoTR04_55 and StoTR05_180 on 2S; centromeric colocalization of the StoTR02_7_tel, StoTR04_55, StoTR06_159, StoTR03_178, StoTR05_180; centromeric colocalization of the telomeric repeats and all StTRs except StoTR01_86; telomeric repeats on the both termini regions |
| 3 | Subtelomeric colocalization of the the StoTR02_7_tel and StoTR04_55 on 3S; interstitial colocalization of the DAPI band and StoTR01_86 on 3S; paracentromeric colocalization of StoTR03_178 on 3S; centromeric colocalization of StoTR02_7_tel, Sto_5S, StoTR03_178 and StoTR05_180. |
| 4 | Interstitial colocalization of the DAPI bands and StoTR01_86 on 4S; paracentromeric StoTR03_178 on 2S; paracentromeric colocalization of the StoTR02_7_tel and StoTR04_55; centromeric colocalization of the StoTR02_7_tel, StoTR04_55, StoTR03_178 and StoTR05_180. |
| 5 | Interstitial colocalization of the the StoTR02_7_tel and StoTR04_55; Interstitial StoTR05_180 on 5S; paracentromeric colocalization of StoTR04_55 and StoTR01_86 on 2S; centromeric colocalization of the StoTR02_7_tel, StoTR04_55, StoTR03_178, StoTR05_180. |
| 6 | Interstitial colocalization of the StoTR02_7_tel, StoTR04_55, StoTR01_86 and StoTR05_180 on 6L; centromeric colocalization of the StoTR02_7_tel, StoTR04_55, StoTR03_178 and StoTR05_180. |
| 7 | Interstitial colocalization of StoTR04_55, StoTR03_178, StoTR05_180 on 7L; paracentromeric colocalization of StoTR04_55 and StoTR01_86; centromeric colocalization of the StoTR02_7_tel, StoTR04_55, StoTR01_86, StoTR03_178 and StoTR05_180. |
| 8 | Interstitial StoTR04_55 and StoTR05_180on 8L; intercalary colocalization of the telomeric repeats and StoTR04_55; paracentromeric colocalization of StoTR04_55 and StoTR01_86; centromeric colocalization of the StoTR02_7_tel, StoTR04_55, StoTR03_178 and StoTR05_180. |
| 9 | Centromeric colocalization of the StoTR02_7_tel, StoTR04_55, StoTR06_159, StoTR03_178 and StoTR05_180. |
| 10 | Interstitial colocalization of the StoTR02_7_tel, StoTR04_55, StoTR06_159; paracentromeric colocalization of DAPI band and StoTR01_86; centromeric colocalization of the StoTR02_7_tel, StoTR04_55, StoTR03_178 and StoTR05_180. |
| 11 | Paracentromeric StoTR04_55 on 10L; weak intercalary StoTR01_86 on 10S; centromeric colocalization of the StoTR02_7_tel, StoTR04_55, StoTR01_86 StoTR03_178 and StoTR05_180. |
| 12 | Subtelocentric Sto_5S on 12S; pericentromeric colocalization of the StoTR02_7_tel, StoTR04_55 and StoTR03_178. |
| 13 | Minor interstitial StoTR05_180 on 12L; intercalary colocalization of the StoTR02_7_tel and StoTR04_55; paracentromeric colocalization of the DAPI band and StoTR01_86; centromeric colocalization of the StoTR02_7_tel, StoTR04_55, StoTR01_86 StoTR03_178 and StoTR05_180. |
